# Adhesive contact of rough brushes

**DOI:** 10.3762/bjnano.9.225

**Published:** 2018-09-07

**Authors:** Qiang Li, Valentin L Popov

**Affiliations:** 1 Berlin University of Technology, 10623 Berlin, Germany; 2 National Research Tomsk State University, 634050 Tomsk, Russia; 3 National Research Tomsk Polytechnic University, 634050 Tomsk, Russia

**Keywords:** adhesion, brushes, contact splitting, pressure sensitive adhesion, roughness

## Abstract

The adhesive contact between a rough brush-like structure and an elastic half-space is numerically simulated using the fast Fourier transform (FFT)-based boundary element method and the mesh-dependent detachment criterion of Pohrt and Popov. The problem is of interest in light of the discussion of the role of contact splitting in the adhesion strength of gecko feet and structured biomimetic materials. For rigid brushes, the contact splitting does not enhance adhesion even if all pillars of the brush are positioned at the same height. Introducing statistical scatter of height leads to a further decrease of the maximum adhesive strength. At the same time, the pull-off force becomes dependent on the previously applied compression force and disappears completely at some critical roughness. For roughness with a subcritical value, the pressure dependence of the pull-off force qualitatively follows the known theory of Fuller and Tabor with moderate modification due to finite size effect of the brush.

## Introduction

The study of adhesive contacts has been largely enhanced by studies of the extremely effective adhesion pads of geckos [1]. For example, the adhesion can be optimized by controlling the size and shape of the fiber cap [2–3]; this mushroom-shaped microstructure can provide a stronger adhesive performance than the flat punch [4–5]. The compliant fiber is known to increase the strength of adhesion [6–7]. Almost all works in this field are based on the idea that contact splitting is the sole reason for the enhanced adhesion [8–9]. In a previous work, we shared a contrary opinion [10]: the *contact splitting alone does not lead to enhancement of adhesion*. The physical reason for this is the macroscopic (on the scale of the whole system) concentration of stress in the vicinity of the boundary of the “apparent contact”. In the present paper we extend the previous work by considering “rough brushes”. Related problems have been studied using a number of purely statistical models, which did not consider the elastic interactions between asperities. Zhuravlev proposed a model (originally published in 1940, whereby the work was later translated into English) consisting of asperities in the form of elastic spheres having the same radius but placed at various heights [11]. Kragelsky presented (originally in 1948) an alternative model of a rough surface as a collection of elastic rods and assumed that the rod heights have a Gaussian distribution [12]. In the classical work of Greenwood and Williamson in 1966, they considered both the exponential and Gaussian distribution of asperity heights [13]. A detailed review of hierarchical models of rough surfaces can be found in [14]. A very similar problem was studied by Fuller and Tabor [15] as early as in 1975.

Contrary to the above mentioned works, we consider the numerically exact solution of the adhesive contact problem using the boundary element method as described in [16] using the mesh-dependent detachment criterion [17], which later was extended to power-law-graded media [18] and extensively tested and validated experimentally in [19]. In this work, we find the dependence of the adhesive force on the size of the brush, the fill factor of pillars and the statistical distribution of the pillar heights (simulating the relative roughness of surfaces in contact). We will show that the adhesion of statistical brushes can be described by a small number of simple analytical dependencies based both on Kendall’s theory of flat-ended stamps [20] and the Fuller and Tabor theory of adhesive contacts [15].

## Modeling

### Model description and main governing parameters

**Figure 1:**
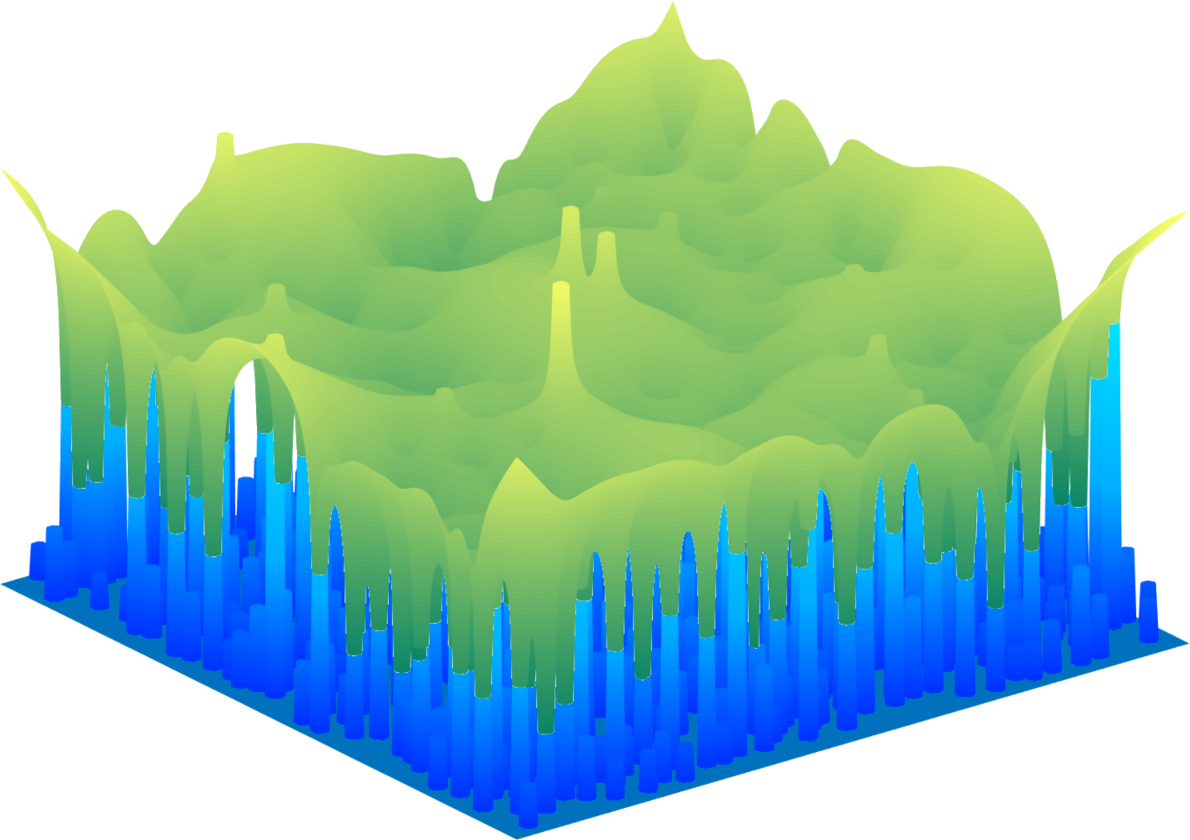
Simulated surface of a rough brush (blue) in adhesive contact with an elastic half-space (green). Along the boundary of the square, one can see the pillars, whose heights are statistically distributed. The elastic half-space is represented only by its surface. At the location of the highest pillars, one can see the “spikes”, which stem from pillars which are strongly pressed into the elastic half-space. At lower pillar heights (see the side of the contact) one can see the “negative spikes” which stem from the not-yet-destroyed adhesive contacts of individual pillars loaded in tension.

We consider a square brush – a rigid body consisting of a large number of cylindrical pillars filling a square area of *A*_0_ = *L* × *L* in contact with an elastic half-space with an example shown in Figure 1. All pillars had the same radius, α = 0.01*L*. The brush is shown in blue while the green color map shows the surface deformation of the elastic half-space during pull-off.

It is known that in the approximation of independent asperities, adhesion can be described in a most general and elegant way if the distribution of asperity heights is described by the exponential probability density,

[1]
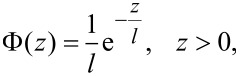


where *l* is the characteristic “roughness”, and *z* the height of an individual pillar. 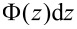 is the probability of finding a pillar with the height between *z* and *z* + d*z*. For easier comparison with existing theoretical predictions, we used this probability distribution throughout the paper.

**Figure 2:**
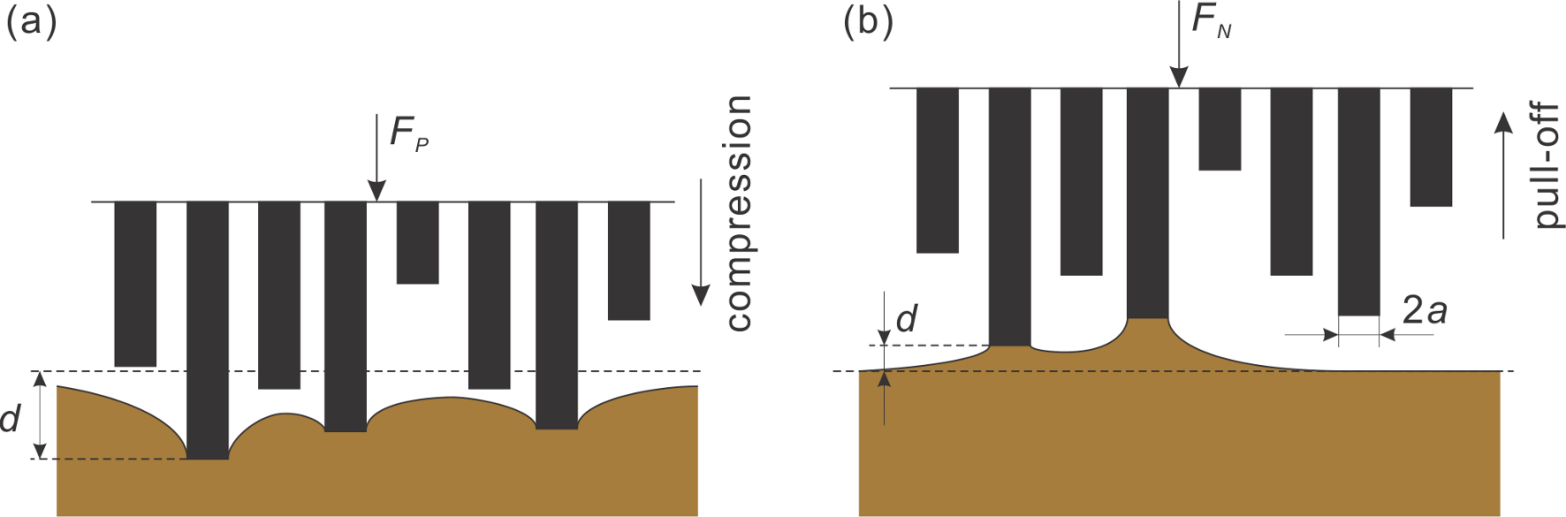
The scheme of indenting and pull-off stages of an adhesive contact of exponentially distributed pillars.

We simulated the following experiment: The brush was first pressed against the elastic half-space with the normal force, *F*_p_ (Figure 2a) and then pulled off as shown in Figure 2b up to complete loss of contact.

The numeric experiment was carried out under conditions of displacement control. If the surface exhibits macroscopic adhesion, the normal force at the moment of complete detachment will be negative; its absolute value is called the force of adhesion, *F*_A_. Let us introduce some characteristic quantities which can be used for comparison with results for brushes:

1. A natural reference force to compare with is the adhesion force of a complete flat-ended square indenter with the size *L* × *L*. In [19], it was argued analytically and confirmed numerically that it can be well-approximated with Kendall’s equations for a cylindrical stamp [20]:

[2]
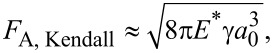


where E* = *E* / (1 − ν^2^) is the effective elastic modulus, *E* is Young’s modulus, ν is Poisson’s ratio, γ is the work of separation (work of adhesion) per unit area, and

[3]
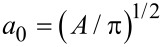


is an effective radius of the square, defined so that the area of a cylinder with the radius *a*_0_ is equal to the area of the square. Note that the maximum adhesive force for a flat-ended square indenter is slightly larger than that predicted by Equation 2.

2. As shown already in [19] and confirmed by detailed simulations in [10], the detachment of a *flat* brush structure (without height distribution), occurs very similar to that of a continuous square, while the force of adhesion can be approximated by

[4]
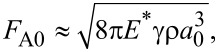


and the fill factor,

[5]
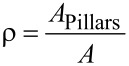


is defined as the ratio of the area filled by pillars to the total apparent area of the square. Equation 4 has a simple physical meaning: it just says that in the case of the not-completely-filled square, the work of adhesion has to be replaced through the effective work of adhesion, γρ. The force of adhesion of a flat brush is a natural reference for comparison with rough brushes.

3. For characterization of the role of the statistical distribution of the pillar height, we consider the critical separation, the point at which the adhesive contact of one single pillar with the radius *a* is lost. This critical separation has been obtained by Kendall [20] as

[6]
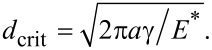


It is clear that if the roughness, *l*, of the brush is much smaller than *d*_crit_, then the brush can be considered as being smooth, while in the opposite case of *l* >> *d*_crit_, the adhesion will be practically completely destroyed by the roughness. We thus anticipate that the parameter *l* / *d*_crit_ will essentially govern the adhesive properties of the rough brush.

### Contact with homogeneous elastic half-space

**Figure 3:**
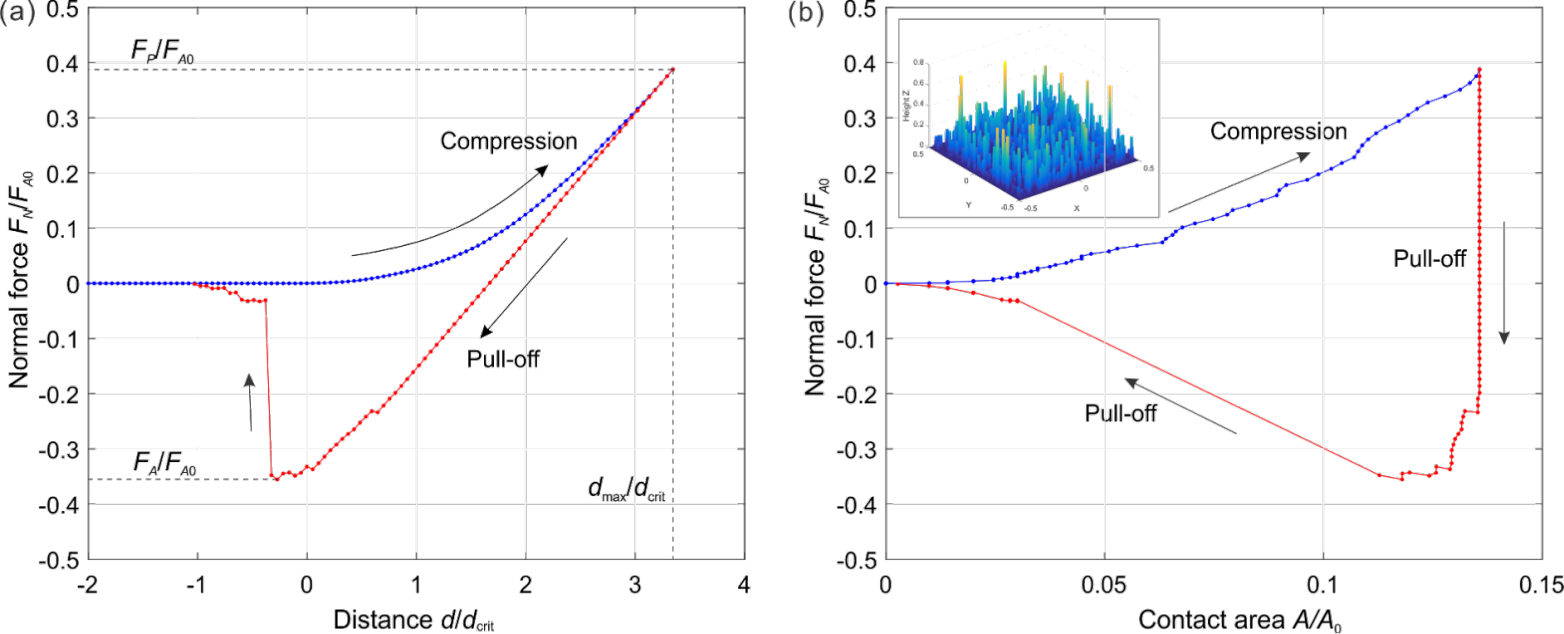
An example of a pillar structure in compression and pull-off contact: (a) load–distance relation; (b) load–contact area relation. Parameters used in this simulation were: Number of pillars: 1000, filling factor: ρ = 0.3, normalized roughness: *l* / *d*_crit_ = 0.42. (a) also provides the exact definition of the preliminary compression force, *F*_p_, and the force of adhesion, *F*_A_.

Figure 3 shows an example of the whole loading cycle starting with indenting the brush into the half-space and following pull-off. The quantities which we are interested in, and which will be presented in the following diagrams, are solely the maximum force during the indentation stage, *F*_p_, and the force of adhesion, *F*_A_, defined as the absolute value of the minimum (negative) value of the normal force during the pull-off stage.

**Figure 4:**
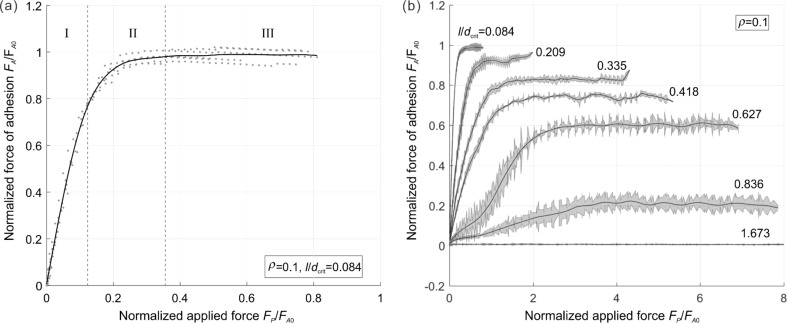
(a) Relation between the compressive force and adhesive force; (b) relation for different values of *l* / *d*_crit_.

In the case of a rough brush, it is clear that the number of pillars that will come into contact with the counter-body depends on the applied normal force, *F*_p_. Correspondingly, the force of adhesion will depend on the preliminary applied normal force. This dependence of the force of adhesion on the applied force is the main characteristic of the brush. An example of such dependence is shown in Figure 4a for the case of a very small roughness parameter *l* / *d*_crit_ = 0.084. In this case, the number of pillars in contact does increase until all pillars are in contact. Due to the small roughness parameter, the adhesive strength in this final state is practically the same as that of a flat brush. The characteristic parts of the curve observed in this case are common for all other cases: The adhesive force first increases linearly with applied force (due to the increasing number of pillars coming into contact). In Figure 4a, we denote this part as region I. Under further increase of the compression force, the force of adhesion finally achieves a plateau, labeled as region III in Figure 4a. Between these regions there is of course some transition, region II. Within the two characteristic regions, the dependence of the adhesion force on the applied force can be written in the form

[7]
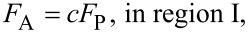


[8]
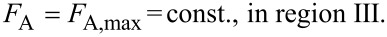


With an increasing roughness, the slope of the linear part of dependence (in region I) becomes smaller and the maximum achievable force of adhesion (value at the plateau) decreases and finally vanishes completely (Figure 4b).

**Figure 5:**
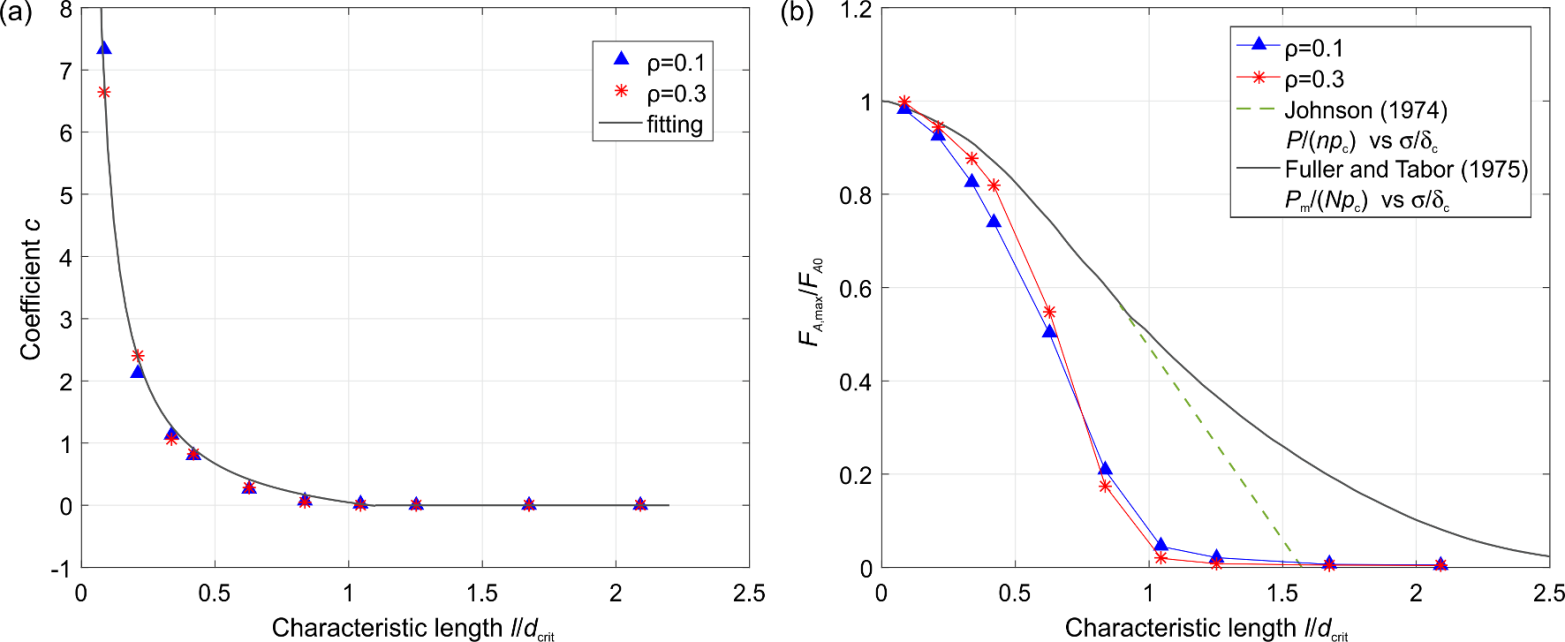
(a) Dependence of the coefficient *C* on the characteristic length in region I (Figure 4); (b) the maximum value of the adhesive force at the plateau (region III, Figure 4).

In region I, a pressure sensitive adhesion must be considered. In this region, the force of adhesion is proportional to the applied normal force and is uniquely determined by the proportionality coefficient, *c*, which sometimes is called the adhesion coefficient [21–22]. The numerically found dependency of the adhesion coefficient on the normalized roughness is shown in Figure 5a. In the approximation of elastically independent pillars, the value of the coefficient of adhesion was found in [21] (see Problem 5 in Chapter 7) to be *c* = *F*_A_ / *F*_p_ = *d*_crit_ / *l* −1. In analogy with this equation, we can try to approximate the numerical result by the equation

[9]
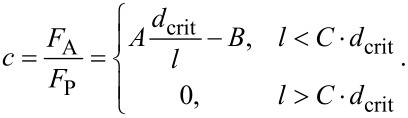


The best fit is achieved with the coefficients *A* = 0.6222, *B* = 0.5758 and *C* = 1.1. Note that dependency of the adhesion coefficient on the dimensionless roughness do not depend on the fill factor (simulation points corresponding to the fill factors 0.1 and 0.3 are fitted by the same curve).

The second important adhesion property of the brush is the maximum adhesion force at the plateau. The dependence of the maximum adhesion force on the normalized roughness is shown in Figure 5b. It also decreases with increasing *l* / *d*_crit_ and practically vanishes at roughness values larger than approximately *l* / *d*_crit_ > 1.1. Again, in a dimensionless presentation, the dependence is only very weakly sensitive to the fill factor and can be considered, to a good approximation, as universal “master curves”. The results by Johnson in 1974 for an exponential distribution of asperity heights (solid black line) [23], and the results by Fuller and Tabor for a Gaussian asperity height distribution (green dashed line) [15] are added in Figure 5b for comparison, where the relative pull-off forces *P* / *np*_c_ and *P*_m_ / *Np*_c_ are plotted against σ / δ_c_, where *P* is the tensile force per unit area, *n* the number of asperities per unit area, *N* the asperity density, *p*_c_ the critical adhesive force of an individual sphere, σ the standard deviation of the distribution of asperity heights, and δ_c_ the critical separation similar to Equation 6 but for spherical asperity.

### Contact with power-law-graded media

In the previous sections, we considered the adhesive contact of brushes that were placed in contact with a homogeneous linear elastic medium. Many biological materials such as skin, bones or bamboo trees are, however, non-homogeneous. This may have a significant impact on the adhesive properties as a softer surface layer may help create an intimate contact with rough surfaces, while the stiffer interior supports a higher final adhesive strength. In this way, properties can be achieved which are not possible for homogeneous materials [24–25].

In the present section, we only consider materials whose elastic coefficient is a function of the normal coordinate *E* = *E*(*z*). This dependence can be either stepwise (as, e.g., in layered or coated materials) or continuous (functionally graded material). For simplicity, we confine ourselves to the model case of materials with a power-law dependence of the elastic modulus on depth, such as

[10]
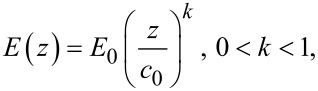


where *E*_0_ is a characteristic elastic modulus and *c*_0_ is a characteristic length. We additionally assume that the Poisson’s ratio of the graded medium is constant and consider only a positive *k*, which means that the material is softer at the surface and stiffer in deeper regions. We also assume the Poisson’s ratio to be constant at ν = 0.3.

As in the previous section, we normalize the roughness to the maximum elongation at the moment of detachment in the contact of a single pillar [26–27]

[11]
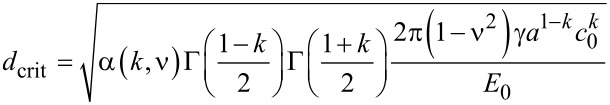


and the adhesive force for that of the flat brush as

[12]
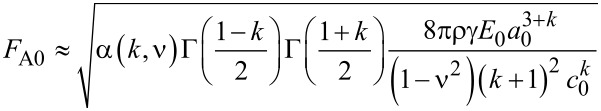


where α and β are

[13]
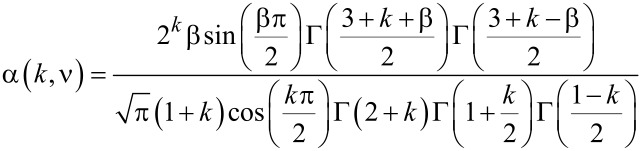


**Figure 6:**
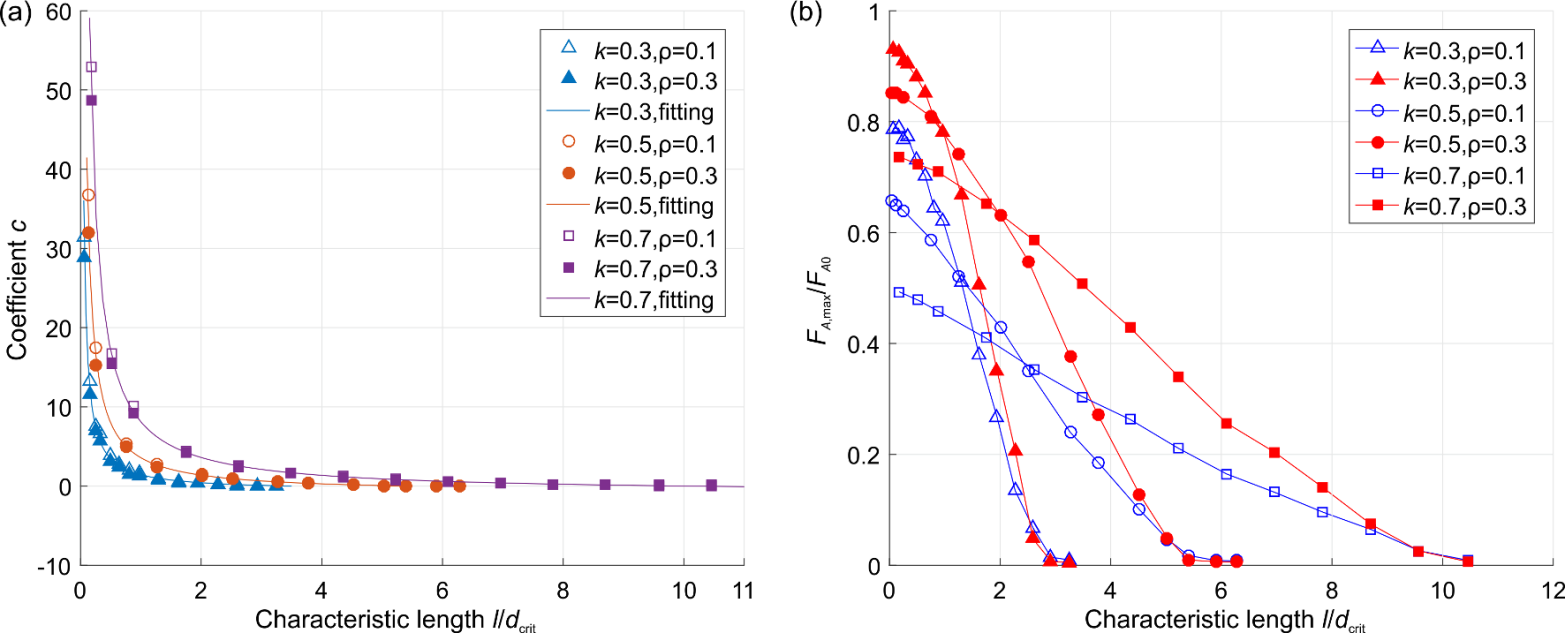
(a) Dependence of adhesion coefficient on the normalized roughness in region I (Figure 4) for different exponents *k*; (b) the maximum value of adhesive force at the plateau (region III, Figure 4) for different exponents *k*.

[14]
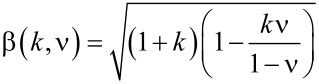


and Γ is the gamma function 
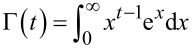
. We carried out simulations for three gradient materials: with the exponent *k* = 0.3, 0.5, and 0.7 and *c*_0_ = 10*a*. All qualitative features found in the case of the contact of a brush with homogeneous materials remain valid also for gradient media. In particular, the force of adhesion increases linearly with the compression force at small forces and achieves a plateau at larger compression forces. Thus, Equations 7–9 are also valid in these cases. The adhesion coefficient in the linear region and the plateau values are shown in Figure 6 for different exponents *k* and fill factors. From Figure 6a it can be seen that the adhesion coefficient is independent of fill factor, and the corresponding universal values of constants are listed in Table 1. However, for large *k*, the required adhesive force for separation increases. However, in the plateau region, the adhesive force decreases with the power *k* and fill factor ρ (Figure 6b). Here one should note that the *F*_A0_ in the normalization is different for different exponents *k*.

**Table 1:** Values of the coefficient *A*, *B* and *C* in the linear region.

	*k* = 0	*k* = 0.3	*k* = 0.5	*k* = 0.7

*A*	0.6222	2.06	4.399	8.999
*B*	0.5758	0.5785	0.816	0.8918
*C*	1.1	3.5	5.4	10.1

## Discussion

Simulations show that the roughness of a brush has two main effects: (1) the pull-off force becomes pressure-dependent and (2) the maximum achievable adhesion force decreases with roughness. Furthermore, there exists a critical roughness at which the macroscopic adhesion disappears completely. In the initial region of pressure-dependent adhesion, the force of adhesion can be characterized by the adhesion coefficient in Equation 9, and the force of adhesion can be easily written in an explicit form:

[15]
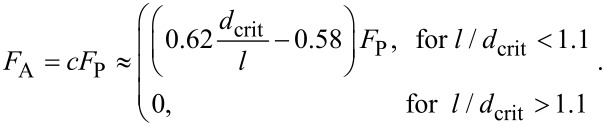


The factors determining the force of adhesion are thus: elastic modulus E*, work of adhesion γ, size of the contact *a*_0_ and normalized roughness *l* / *d*_crit_.

In the case of power-law-graded materials, the situation may change significantly. The main qualitative difference can be observed in the critical roughness, at which the macroscopic adhesion disappears completely. While for the homogeneous material, adhesion vanishes when the roughness is of the order of the critical length *d*_crit_ for single pillar; for the medium with *k* = 0.7, the critical roughness becomes ten times larger than the critical length *d*_crit_. Interestingly, while the maximum normalized force of adhesion in the case of graded media clearly depends on the fill factor, the critical roughness at which the adhesion vanishes seems not to depend on the fill factor and is universally determined by the grading exponent *k*.

Thus, we conclude that material gradients with a positive grading exponent *k* strongly enhance adhesion to very rough surfaces.

## Conclusion

Numerical simulations of finite brushes using the boundary element method show that the earlier simplified analysis by Fuller and Tabor can still be used if corrected by multiplicative factors of the order of unity. These factors have been determined numerically. Unlike the statistical models developed previously, the elastic interactions between the pillars were taken into account in this study using a numerically exact solution with the boundary element method.

We found that for weak compression, the adhesive force is proportional to the applied load and becomes constant at larger normal forces. Adhesion completely vanishes if the roughness is larger than the critical length of detachment for a single pillar of the brush. Similar regularities are valid for graded materials. However, the critical value of roughness may now strongly exceed the critical detachment length for one pillar.
